# Pancreaticopleural Fistula Causing Massive Right Hydrothorax and Respiratory Failure

**DOI:** 10.1155/2016/8294056

**Published:** 2016-09-22

**Authors:** Esther Ern-Hwei Chan, Vishalkumar Girishchandra Shelat

**Affiliations:** Department of General Surgery, Tan Tock Seng Hospital, Singapore

## Abstract

Hydrothorax secondary to a pancreaticopleural fistula (PPF) is a rare complication of acute pancreatitis. In patients with a history of pancreatitis, diagnosis is made by detection of amylase in the pleural exudate. Imaging, particularly magnetic resonance cholangiopancreatography, aids in the detection of pancreatic ductal disruption. Management includes thoracocentesis and pancreatic duct drainage or pancreatic resection procedures. We present a case of massive right hydrothorax secondary to a PPF due to recurrent acute pancreatitis. Due to respiratory failure, urgent thoracocentesis was done. Distal pancreatectomy with splenectomy and cholecystectomy was performed. The patient remains well at one-year follow-up.

## 1. Introduction

Extra-abdominal complications of acute pancreatitis such as a hydrothorax secondary to a pancreaticopleural fistula (PPF) are extremely rare. It is reported to occur in approximately 0.4% of patients with pancreatitis, although the precise incidence is unknown [1]. The diagnosis is often delayed as many pulmonary investigations are often performed before the final diagnosis of a PPF is reached. The classical finding which points towards a diagnosis of PPF is a high amylase level in pleural fluid. Early surgical intervention can prevent recurrent pleural effusions and negate the need for multiple thoracocentesis. We report a rare case of a massive right-sided hydrothorax and respiratory failure on a background of a PPF.

## 2. Case Presentation

A 51-year-old gentleman was admitted with a 2-day history of right-sided abdominal pain. There was associated nausea, but no vomiting, diarrhoea, or fever. He had a background of hypertension, hyperlipidaemia, ischaemic heart disease, a previous stroke, and a previous gastric perforation which was managed nonoperatively. He was a chronic smoker with a 20-pack-year history and was an ex-alcoholic, drinking 5 bottles of beer a day, stopping 9 months prior. He had two previous pancreatitis-related admissions, with the most recent admission 7 months prior. Ultrasound of the abdomen did not reveal gallstones. Lipid panel and calcium panel were unremarkable; hence, alcohol was adjudged to be the aetiological factor for his recurrent acute pancreatitis.

**Figure 1:**
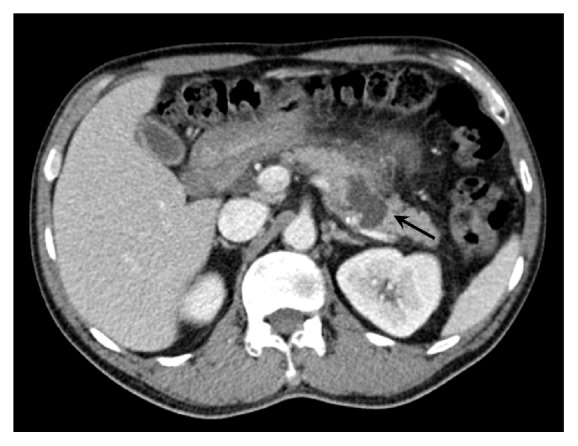
4.1 cm pancreatic pseudocyst in distal pancreatic body (marked by arrow).

At the previous admission for acute-on-chronic pancreatitis, a computed tomography (CT) scan of the abdomen revealed a 4.1 cm pancreatic pseudocyst in the distal pancreatic body (Figure 1). Ca19-9 was 14 units/mL and serum amylase levels were 160 units/L. He made an uneventful recovery and was discharged after 2 days. A month later, he had an outpatient endoscopic ultrasound (EUS) which revealed a 27 mm pseudocyst in the distal pancreas.

**Figure 2:**
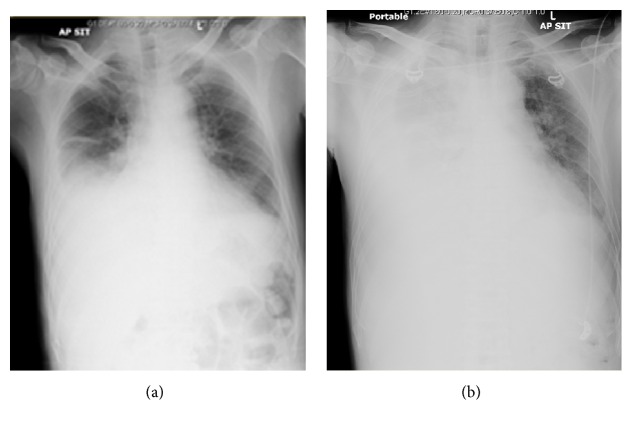
Chest radiographs showing a right pleural effusion on admission (a) with worsening of the effusion one day after (b).

At this current admission, a chest radiograph on admission revealed consolidation in the right lower zone associated with an ipsilateral pleural effusion (Figure 2). He did not have any shortness of breath and had good oxygen saturations; hence, no intervention was required at this time. However, the next day he was unable to maintain oxygen saturations and required increasing amounts of supplemental oxygen. The patient was noted to be in Type 1 respiratory failure on arterial blood gas and chest radiograph showed worsening of the right pleural effusion (Figure 2). Ultrasound-guided percutaneous drainage of the pleural effusion drained 1000 mL of haemoserous fluid. Biochemical analysis of the pleural fluid revealed it to be exudative with a fluid amylase level of >2000 units/L. Fluid cultures grew* Klebsiella pneumoniae* and a culture-directed course of antibiotics was prescribed. In view of the high pleural fluid amylase levels, a diagnosis of a PPF was made and he was given octreotide 100 mcg 8-hourly subcutaneously for 5 days. The pleural drain was left in situ for 7 days and it drained 2.5 litres of haemoserous fluid in total prior to removal.

The patient subsequently underwent magnetic resonance cholangiopancreatography (MRCP) to delineate PPF anatomy. A retroperitoneal collection communicating with the distal main pancreatic duct was evident. Dilated extrahepatic and intrahepatic bile ducts with a focal stricture in the distal common bile duct (CBD) were also noted. The liver function test was normal. In view of these findings, the case was discussed at the Hepatopancreatobiliary Multidisciplinary Conference. The conference recommended EUS with fine needle aspiration (FNA) to rule out malignancy as the cause of the biliary stricture. EUS revealed a large periampullary diverticulum to be the cause of the CBD dilatation. No strictures or filling defects were noted in the CBD and there was no head of pancreas lesion. Gallbladder adenomyomatosis was also noted.

He completed 2 weeks of intravenous antibiotics and oral antibiotics were continued until the day of surgery. Surgery was planned after 4 weeks. Due to ongoing antibiotic treatment, a decision was made to administer splenectomy vaccinations postoperatively. Elective open distal pancreatectomy with splenectomy and cholecystectomy was performed. Total operative time was 3.5 hours. The patient made an uneventful recovery and was discharged home on postoperative day 5 with postsplenectomy vaccinations administered at 1-month follow-up. Histology revealed chronic cholecystitis, chronic pancreatitis, and a pancreatic pseudocyst with haemorrhage. No malignancy was seen. He remains well at 12 months' follow-up.

## 3. Discussion

Massive pleural effusion caused by PPF is a very rare complication of pancreatitis, most commonly associated with alcoholic chronic pancreatitis [2]. A PPF results from disruption of a major pancreatic duct, usually due to underlying pancreatic disease, trauma, or iatrogenic injury. It is proposed that a ductal disruption on the anterior surface of the pancreas usually leads to pancreatic ascites, whereas a ductal disruption on the posterior surface may result in thoracic fluid collections as the fluid spreads retroperitoneally through pathways of least resistance at the aortic or oesophageal hiatus [3]. It remains elusive if the fluid transgresses via lymphatic channels or directly through the hiatus or via diaphragmatic microperforations. In cases of a PPF resulting in a hydrothorax, approximately 75% occur on the left side, although they may also be right-sided or bilateral [4]. In patients presenting with pleural effusions without recent abdominal symptoms, reaching the diagnosis of a PPF may pose a diagnostic challenge. The characteristic feature of a PPF is an exudative pleural fluid with high amylase content. In our patient, the fluid was sent for amylase testing immediately upon drainage.

Once a diagnosis of a PPF has been made, imaging is useful in identifying the cause and delineating the anatomy and location of the fistulous tracts. Contrast-enhanced computerized tomography (CT) scan is the standard modality for the evaluation of acute pancreatitis and its complications such as necrosis or pseudocyst formation; however, it is not sensitive in assessing the presence and morphology of fistulous tracts, which is better visualised on MRCP [5]. The triad of (a) history of acute pancreatitis (b) imaging showing pancreatic ductal disruption with pleural effusion and (c) amylase rich pleural exudate is diagnostic of pancreatic hydrothorax.

Thoracocentesis not only aids in diagnosis by obtaining fluid for biochemistry, but also alleviates pulmonary symptoms. Reduced stimulation of pancreatic exocrine secretions reduces fistula output and can be achieved with somatostatin analogues. Endoscopic retrograde cholangiopancreatography (ERCP) is diagnostic and potentially therapeutic in cases of PPF. Therapeutic effect is achieved by transpapillary pancreatic duct stent placement to bridge the areas of ductal disruption to decrease the pancreatic ductal pressure and allow for the fistula to seal [6]. It is a less invasive alternative to surgery, although a significant proportion of patients undergoing ERCP still ultimately require surgical intervention [2]. In patients with recurrent idiopathic pancreatitis, ERCP may aid in the diagnosis of rare aetiologies [7]. A multidisciplinary approach in diagnosing pancreatic pseudocysts is essential as splenic artery pseudoaneurysm can mimic a pseudocyst on CT and MRCP [8]. In patients with pancreatic necrosis, a minimal invasive approach is proposed, but for PPFs a distal pancreatectomy with splenectomy is recommended [1, 9, 10]. Other operative procedures recommended are pancreatic duct anastomosis with an intestinal loop (the Partington-Rochelle procedure), pancreaticoduodenectomy, cystogastrostomy, and cystojejunostomy [1, 4, 9]. Choice of operative procedure will depend on the anatomy of the PPF; as in our patient, distal ductal disruptions are dealt with by distal pancreatic resections. Pleural effusions tend to be recurrent despite repeated thoracocentesis, hence the recommendation by King et al. [4] to manage PPFs with early surgical resection. Laparoscopic distal pancreatectomy remains the gold standard; however, in our patient we anticipated a difficult operative course due to multiple episodes of acute pancreatitis and previous gastric perforation, hence the decision for open surgery. We also performed splenectomy as (a) it reduces blood loss due to control of splenic vessels and (b) adult immunocompetent patients rarely manifest with opportunistic postsplenectomy sepsis [11].

## 4. Conclusion

PPF is a rare complication of recurrent acute pancreatitis and presentation with a right-sided hydrothorax is even rarer. Pleural fluid amylase levels should be tested in all patients with a history of pancreatitis presenting with a hydrothorax. The triad of a history of pancreatitis, imaging demonstrating pancreatic ductal disconnection, and amylase rich pleural exudate establishes the diagnosis of pancreatic hydrothorax. Surgical intervention is the definitive treatment option.
